# Screening Accuracy and Clinical Application of the Brief Infant-Toddler Social and Emotional Assessment (BITSEA)

**DOI:** 10.1371/journal.pone.0072602

**Published:** 2013-08-30

**Authors:** Ingrid Kruizinga, Wilma Jansen, Cathelijne L. Mieloo, Alice S. Carter, Hein Raat

**Affiliations:** 1 Department of Public Health, Erasmus University Medical Center, Rotterdam, The Netherlands; 2 Department of Youth Policy, Rotterdam Municipal Health Service (GGD Rotterdam- Rijnmond), Rotterdam, The Netherlands; 3 Department of Psychology, University of Massachusetts Boston, Boston, Massachusetts, United States of America; The University of Queensland, Australia

## Abstract

**Background:**

The Brief Infant-Toddler Social and Emotional Assessment (BITSEA) is a promising questionnaire for the early detection of psychosocial problems in toddlers. The screening accuracy and clinical application were evaluated.

**Methods:**

In a community sample of 2-year-olds (N = 2060), screening accuracy of the BITSEA Problem scale was examined regarding a clinical CBCL1.5-5 Total Problem score. For the total population and subgroups by child’s gender and ethnicity Receiver Operating Characteristic (ROC) curves were calculated, and across a range of BITSEA Problem scores, sensitivity, specificity, likelihood ratio’s, diagnostic odds ratio and Youden’s index. Clinical application of the BITSEA was examined by evaluating the relation between the scale scores and the clinical decision of the child health professional.

**Results:**

The area under the ROC curve (95% confidence interval) of the Problem scale was 0.97(0.95–0.98), there were no significant differences between subgroups. The association between clinical decision and BITSEA Problem score (*B* = 2.5) and Competence score (*B* = −0.7) was significant (p<0.05).

**Conclusions:**

The results indicate that the BITSEA Problem scale has good discriminative power to differentiate children with and without psychosocial problems. Referred children had less favourable scores compared to children that were not referred. The BITSEA may be helpful in the early detection of psychosocial problems.

## Introduction

Preventive child health care has always focused on the detection of physical conditions. Recently, its focus has been expanded to mental health issues [1], offering an opportunity for the early detection of psychosocial problems among preschool children. In the Netherlands, preventive child health care for young children is delivered through community well-child clinics that are free of charge and provide routine developmental assessment and vaccinations [2]. One approach for facilitating early recognition and identification of psychosocial problems is to use parent-completed questionnaires as part of routine primary care visits (i.e. well-child visits) [3]. Early detection instruments of psychosocial problems, intended for use in preventive child health care, should have adequate psychometric properties (i.e. reliability and validity), and should also be short, easy to administer, score, and interpret [4], [5]. Furthermore, early detection instruments should be able to correctly discriminate children with and without psychosocial problems. Of course early detection will not be without errors, but should be as accurate as possible as to minimize the expenses associated with over-referrals and under-detection [4]. The identification of cutpoints and their concomitant accuracy is therefore important, since this enables child health professionals to determine how many responses that indicate psychosocial problems, reliably indicating the actual presence of psychosocial problems. When cutpoints are identified, indices for screening accuracy (e.g. sensitivity and specificity) can be established for the questionnaire.

In the setting of preventive child health care general early detection instruments are warranted, since the aim is to early detect a broad range of possible psychosocial problems. The Child Behavior Checklist 1.5–5 (CBCL1.5–5) [6] and Infant-Toddler Social and Emotional Assessment (ITSEA) [7], [8] are early detection instruments that are well-validated and measure a broad range of psychosocial problems, and in the case of the ITSEA also delays in competencies. However both instruments are too extensive to apply in the context of well-child visits. The ITSEA has been reported to have adequate psychometric properties [7], [8], and exists in a shorter version; the Brief Infant-Toddler Social and Emotional Assessment (BITSEA). The BITSEA comprises 42 items that measure psychosocial problems as well as delays in the acquisition of competencies in toddlers (1–3 year olds). Previous studies have shown that the BITSEA has adequate reliability and validity [9]–[11].

Previous studies have evaluated the sensitivity and specificity of the BITSEA [9], [12]. One study, performed in Turkey among a community sample of 462 children, examined the sensitivity and specificity of only the BITSEA Competence scale relative to children treated in a child psychiatry outpatient clinic with an autism diagnosis. In this study, the sensitivity was 72%–93% and specificity was 76%–85%, depending on the cutpoint chosen [12]. In another study, performed in the United States of America, the sensitivity and specificity was examined in a community sample of 1280 children. In this study, the BITSEA Problem scale had, relative to the CBCL1.5–5 a sensitivity of 93.2% and a specificity of 78.0%. The BITSEA Competence scale was examined relative to low ITSEA Competence, and had a sensitivity of 68.9% and a specificity of 95.1% [9]. BITSEA Problem scale cutpoints were chosen at scores of ≥75^th^ percentile and for the BITSEA Competence scale cutpoints were chosen at scores of <15^th^ percentile [13].

In the present study we aim to evaluate the screening accuracy more extensively than prior studies. The screening accuracy will be evaluated with multiple indices (i.e. area under the curve, sensitivity, specificity, likelihood ratio’s, diagnostic odds ratio and Youden’s index) by calculating receiver operating characteristic (ROC) curves of the BITSEA Problem scale relative to a CBCL Total Problem score in the clinical range. In our study we present indices of screening accuracy for a range of BITSEA Problem scores, because different cutpoints might be chosen in different settings (e.g. clinical application versus epidemiological research). The screening accuracy of the BITSEA Competence scale was not evaluated with a reference group of children with a CBCL Total Problem score in the clinical range, since the CBCL Total Problem score does not measure competencies. Previous studies showed differences in mean BITSEA scores between boys and girls (with boys scoring less favourably) [9]–[11] and between native and non-native children (with non-native children scoring less favourably) [11], therefore the screening accuracy will also be evaluated in subgroups by gender and ethnicity.

Furthermore, we evaluated the clinical application of the BITSEA Problem and Competence scale, by comparing BITSEA scores with the clinical decision of the child health professionals. We hypothesised that the clinical decision of the child health professional is associated with on the one hand higher BITSEA Problem scale scores, as high BITSEA Problem scale scores are expected to be indicative of problems, and on the other hand lower BITSEA Competence scale scores, as low BITSEA Competence scale scores are expected to be indicative of problems (i.e. delays in competencies).

## Methods

### Ethics Statement

Only anonymous data were used and the questionnaires were completed on a voluntary basis by the parents. Parents received written information on these questionnaires and were free to refuse to participation. Observational research with data does not fall within the ambit of the Dutch Act on research involving human subjects [14] and does not require the approval of an ethics review board. Informed consent was obtained for the use of the CBCL, since this data collection was not part of the routine health examinations. The Medical Ethics Committee of the Erasmus Medical Centre Rotterdam declared to have no objection (‘formal waiver’) regarding the study protocol and consent procedures.

### Design and Participants

The data was gathered between April 2010 and April 2011 by child health care organizations in the context of routine health examinations in the Rotterdam area, the Netherlands. Before coming to the well-child visit, parents were invited to complete the BITSEA and CBCL1.5-5. Child health professionals were trained to score the answers given by parents on the BITSEA and use the cutpoint as identified in the US [15] in their assessment whether children are at risk for, or currently experiencing, psychosocial problems. Child health professionals were blind for the answers on the CBCL. Parents of 3170 2-year old children that attended the well-child visit handed in the questionnaire (95.5% of all parents that attended the well-child visit). Of these parents, 2184 (68.9%) parents gave informed consent for participation in the study. Children were excluded from analyses if there were too many missing items on the BITSEA Problem (>5) and Competence (>2) scales [15] (n = 50) and CBCL1.5–5 (>8) (n = 74), leaving a study population of 2060 (94.3%) children (mean age: 23.7 months, SD = 0.7; 49.6% boys, 33.8% non-native). None of the children were under treatment of a mental health professional at the time of inclusion. Details on the design of the study are described elsewhere [11], [16]. The community sample consists of 43 (2.1%) children (mean age: 23.9, SD = 0.7; 51.2% boys, non-native 69.8%) that scored in the clinical range of the CBCL Total Problem score (raw score>60).

### Measures

The BITSEA is designed for children aged 12 months to 36 months and consists of 42 items with three response options (‘not true/rarely’, ‘somewhat true/sometimes’, ‘very true/often’). There are two multi-item scales, a Problem scale (31 items) and a Competence scale (11 items). The Problem scale assesses social-emotional/behavioral problems such as aggression, defiance, overactivity, negative emotionality, anxiety, and withdrawal. The Competence scale assesses social-emotional abilities such as empathy, prosocial behaviors, and compliance [13]. Responses should be summed for each scale, a high score on the Problem scale or a low score on the Competence scale is less favourable [15]. Previous studies showed that the BITSEA has good reliability and validity [9]–[11]. A study in the Netherlands yielded for the BITSEA Problem and Competence scale respectively an internal consistency (Cronbach’s alpha) of 0.76 and 0.63 a test-retest reliability (intraclass correlation, ICC) of 0.75 and 0.61; parent-childcare provider interrater reliability (ICC) of 0.30 and 0.17; and significant positive correlations (Problem scale) and significant negative correlations (Competence scale) with the CBCL1.5–5 Total Problem score [11].

The CBCL1.5-5 is a well-validated [6] 100-item questionnaire designed for children aged 18 months to 5 years and has two domains (Internalising and Externalising) that are combined to give a Total Problem score. Answers are given on a 3-point scale (‘not true’, ‘somewhat or sometimes true’ and ‘very or often true’). Children with a Total Problem score greater than 60, score in the clinical range of the CBCL1.5-5.

Ethnicity of the child was determined based on parental country of birth: a child was considered native if both parents were born in the Netherlands [17].

Clinical decision was measured as the decision of the child health professional whether or not to refer the child to a more specialized mental health professional and/or to request a follow-up consultation, as recorded in the electronic medical file. Hereinafter, we will only mention ‘referral’ as clinical decision, but it also entails the request for follow-up consultation.

### Analyses

#### Mean and median BITSEA scores and CBCL1.5-5 Total Problem score

Differences in mean BITSEA Problem and BITSEA Competence scores and CBCL Total Problem score between subgroups by child gender and ethnicity are tested with independent sample t-tests. Differences in median and distribution of BITSEA Problem and BITSEA Competence scores and CBCL Total Problem score between children with and without a clinical CBCL Total Problem score were tested with a Mann-Whitney U test for the total population and for subgroups by child gender and ethnicity. A Mann-Whitney U test was appropriate since the subgroups were small and the assumption of normality could not be met.

#### Screening accuracy

Screening accuracy of the BITSEA Problem scale was evaluated by calculating receiver operating characteristics (ROC) curves, with a reference group that consists of children with a CBCL Total Problem score in the clinical range. Area under the ROC curve was examined, along with, for a range of BITSEA Problem scale scores; sensitivity, specificity, positive test likelihood ratio (LHR^+^) and negative test likelihood ratio (LHR^−^), diagnostic odds ratio (OR) and Youden’s index. All indices for screening accuracy were evaluated for the total sample as well as for boys and girls and for native and non-native children separately.

The ROC curve is a plot of sensitivity as a function of 1-specificity for all possible cutpoints of the BITSEA. The greater the area under the curve (AUC), the more discriminative power the BITSEA has in differentiating children with and without psychosocial problems. An AUC≥0.90 indicates high accuracy; 0.70≤AUC<0.90 indicates moderate accuracy; 0.50≤AUC<0.70 indicates low accuracy; and AUC = 0.50 is chance level accuracy [18]. We examined the 95% confidence intervals of the AUCs to evaluate whether the screening accuracy differed significantly between subgroups.

The Youden index was calculated, which is defined as the maximum vertical distance between the ROC curve and the diagonal or chance line and is calculated as *Youden’s index = sensitivity+specificity−1*. The higher the Youden index, the more optimal a cutpoint is [19].

Sensitivity is the proportion of true positives that are correctly identified by the test; specificity is the proportion of true negatives that are correctly identified by the test. To further investigate the correctness of classification, likelihood ratios were calculated. *LHR^+^ = sensitvitiy/(1−specificity)* is the ratio of the probability of a positive test result if the outcome is positive (true positive) to the probability of a positive test result if the outcome is negative (false positive); *LHR* ^−^ *= (1−sensitivity)/specificity* is the ratio of the probability of a negative test result if the outcome is positive (false negative) to the probability of a negative test result if the outcome is negative (true negative). LHR^+^>7.00 and LHR^−^<0.30 indicate high screening accuracy [20].

The OR = sensitivity*specificity/((1−sensitivity)*(1−specificity)) = LHR^+^/LHR ^−^ of a test is the ratio of the odds of a positive test result when having the ‘disorder’ relative to the odds of a positive test result when not having the ‘disorder’. The values of OR ranges from zero to infinity, with higher values indicating better discriminatory test performance. OR>20.00 indicate high screening accuracy [20].

The AUC, Youden’s index, sensitivity, specificity, LHR^+^, LHR^−^ and OR are independent of prevalence of the ‘disorder’, as opposed to the positive predictive value and negative predictive value [20].

#### Clinical Application

The clinical application of the BITSEA was explored by evaluating the relation between BITSEA Problem and Competence scores and the decision of the child health professional to refer to another mental health professional.

Because the observations within child health professional were not independent from each other, a multilevel regression analyses was used to evaluate the relation between the clinical decision as independent variable and the BITSEA scale scores as dependent variable, corrected for confounding effects of child’s gender and ethnicity. The effect sizes of the differences in mean BITSEA scale scores between children that were and were not referred were defined as *Cohen’s d = [mean(worried)–mean(not worried)]/SD_worried._* [21] A small effect is defined as 0.20≤*d*<0.50; a medium effect is defined as 0.50≤*d*<0.80; and a large effect is defined as *d*≥0.80. Additionally, frequencies of referral for children scoring in the clinical range of the BITSEA were evaluated. Cutpoints are set at the 25^th^ percentile for the Problem scale; and at 15^th^ percentile for the Competence scale, as is specified in the manual of the BITSEA [15].

Data regarding the clinical decision of the child health professional were available for 1579 (76.7%) children (combined data of 481 (23.3%) children were unavailable due to missing patient-codes and differences in registration methods between child health care organizations). Significant differences between the group with complete data and the group with incomplete data were found only for the mean CBCL Total Problem score (p = 0.04). Similar differences were not observed for mean BITSEA Problem score, mean BITSEA Competence score, age of the child and parents, child gender and ethnicity, country of birth of the parents, person who completed the questionnaire and family composition. The effect size of the significant differences in CBCL Total Problem score between the group with complete data and the group with incomplete data, however, was small and was taken as an indication that the data were ‘missing at random’ (mean CBCL Total Problem score, Cohen’s d = 0.10).

Multilevel regression analyses were performed in SAS software version 9.2 (SAS Institute Inc., 2009). All other analyses were performed in SPSS 19.0 (SPSS Inc. 2010).

## Results

### Mean and Median BITSEA Scores and CBCL1.5-5 Total Problem Score

Mean BITSEA Problem scale score, BITSEA Competence scale score and CBCL Total Problem score and standard deviations for the total population and subgroups by child gender and ethnicity are presented in Table 1. All mean scores differed between boys and girls and native and non-native children (p<0.01), except for the mean CBCL Total Problem score between boys and girls (p = 0.96).

**Table 1:** Means and standard deviations of BITSEA Problem and Competence scores and CBCL Total Problem score for the total population and for subgroups by child gender and ethnicity.

Mean (SD)	Total	Boys^1^	Girls^1^	Native	Non-native
	N = 2060 (100%)	n = 1021 (49.6%)	n = 1033 (50.1%)	n = 1364 (66.2%)	n = 696 (33.8%)
**CBCL Total Problem**	19.1 (15.5)	19.1 (15.5)	19.1 (15.4)	16.8 (13.6)*	23.7 (17.7)*
**BITSEA Problem**	7.7 (5.2)	8.1 (5.4)*	7.3 (4.9)*	6.8 (4.4)*	9.4 (6.1)*
**BITSEA Competence**	17.8 (2.9)	17.5 (2.9)*	18.1 (2.9)*	18.2 (2.6)*	16.9 (3.3)*

1 Percentages do not sum to 100 because of missing values.

* mean scores differed significantly between boys & girls and native & non-native children, p<0.05.

Table 2 presents the median scores and 25 and 75 percentile for children with and without a CBCL Total Problem score in the clinical range, for the total subpopulations as well as for subgroups by child gender and ethnicity. Between the subpopulations of children with and without a CBCL Total Problem score in the clinical range, all distributions and median scores differed significantly (p<0.05), except for the median BITSEA Competence score between girls (p = 0.18) and native children (p = 0.22). Within the subpopulation of children *without* a CBCL Total Problem score in the clinical range, all distributions and median scores differed between subgroups of gender and ethnicity (p<0.01), except for the CBCL Total Problem score distribution (p = 0.96) and median (p = 0.87) between boys and girls. Within the subpopulation of children *with* a CBCL Total Problem score in the clinical range, all distributions and median scores did *not* differ between subgroups of gender and ethnicity (p>0.05), except for the Competence score distribution (p = 0.03) between boys and girls.

**Table 2:** Median and 25–75 percentile of BITSEA Problem and Competence scores and CBCL Total Problem score for children with and without a CBCL Total Problem score in the clinical range, for the total subpopulations and for subgroups by child gender and ethnicity.

		CBCL Total Problem score<clinical range n = 2017 (97.9%)					CBCL Total Problem sore>clinical range n = 43 (2.1%)				
		total	boys^1^	girls^1^	native	non-native	Total	boys^1^	girls^1^	native	non-native
		n = 2017(100%)	n = 999(49.5%)	n = 1012(50.2%)	n = 1351(67.0%)	n = 696(33.0%)	n = 43(100%)	n = 22(51.2%)	n = 21(48.8%)	n = 13(30.2%)	n = 30(69.8%)
**CBCL Total Problem**	mdn	15.0*	15.0*	15.0*	14.0* †	20.0* †	71.0*	71.5*	71.5*	75.0*	69.5*
	25%–75%	7.0–26.0*	8.0–26.0*	7.0–26.0*	6.0–23.0* †	10.0–32.0* †	66.0–76.0*	64.8–78.8*	66.5–75.0*	67.5–77.5*	64.0–75.0*
**BITSEA Problem**	mdn	7.0*	7.0* †	6.0* †	6.0* †	8.0* †	20.0*	19.5*	20.0*	20.0*	21.5*
	25%–75%	4.0–10.0*	4.0–10.0* †	4.0–10.0* †	4.0–9.0* †	5.0–12.0* †	17.0–25.0*	16.8–26.5*	17.0–24.5*	18.0–21.5*	16.0–26.0*
**BITSEA Competence**	mdn	18.0*	18.0* †	19.0†	19.0†	17.0* †	16.0*	15.0*	16.0	16.0	15.5*
	25%–75%	16.0–20.0*	16.0–20.0* †	17.0–20.0* †	17.0–20.0* †	15.0–19.0* †	14.0–18.0*	11.0–17.0* †	15.0–19.0* †	15.5–19.0*	13.5–17.3*

1 Percentages do not sum to 100 because of missing values.

* significant difference in distribution and median scores *between* subpopulations with and without a CBCL Total Problem score in the clinical range, p<0.05.

† significant difference in distribution and median scores *within* subpopulations with and without CBCL Total Problem score in the clinical range, *between* boys & girls and native & non-native children, p<0.05.

### Screening Accuracy

In Table 3 are the AUC and sensitivity, specificity, LHR+, LHR-, OR and Youden’s index presented for a range of BITSEA Problem score cutpoints, for the total population and for subgroups by gender and ethnicity.

**Table 3:** Screening accuracy for a range of BITSEA Problem scores, relative to a CBCL Total problem score in the clinical range.

Total, N = 2060	AUC = 0.97 (95% CI = 0.95–0.98)						
	score	sensitivity	specificity	LHR+	LHR−	OR	Youden’s index
	11	0.98	0.78	4.39	0.03	146.67	0.75
	12	0.98	0.82	5.58	0.03	197.98	0.80
	13	0.95	0.87	7.20	0.05	134.36	0.82
	**14**	**0.95**	**0.90**	**9.38**	**0.05**	**181.20**	**0.85**
	15	0.88	0.92	11.28	0.13	89.42	0.81
	16	0.84	0.94	14.94	0.17	86.65	0.78
	17	0.79	0.96	18.54	0.22	84.82	0.75
**Boys, n = 1021**	AUC = 0.95 (95% CI = 0.92–0.98)						
	11	0.95	0.75	3.89	0.06	64.63	0.71
	12	0.95	0.81	5.05	0.06	90.00	0.77
	13	0.91	0.85	6.18	0.11	57.96	0.76
	**14**	**0.91**	**0.88**	**7.70**	**0.10**	**74.66**	**0.79**
	15	0.82	0.90	8.60	0.20	42.82	0.72
	16	0.82	0.93	12.57	0.19	64.66	0.75
	17	0.77	0.95	14.85	0.24	61.92	0.72
**Girls, n = 1033**	AUC = 0.98 (95% CI = 0.97–0.99)						
	11	1.00	0.80	4.99	0.00	×	0.80
	12	1.00	0.84	6.21	0.00	×	0.84
	13	1.00	0.88	8.50	0.00	×	0.88
	**14**	**1.00**	**0.92**	**11.77**	**0.00**	**×**	**0.92**
	15	0.95	0.94	15.55	0.05	306.45	0.89
	16	0.86	0.95	18.46	0.15	123.19	0.81
	17	0.81	0.97	24.83	0.20	126.08	0.78
**Native, n = 1364**	AUC = 0.98 (95% CI = 0.97–1.00)						
	14	0.92	0.93	13.86	0.08	168.13	0.86
	15	0.92	0.95	19.19	0.08	237.42	0.87
	16	0.92	0.97	29.00	0.08	365.02	0.89
	**17**	**0.92**	**0.98**	**40.23**	**0.08**	**510.97**	**0.90**
	18	0.85	0.98	42.34	0.16	269.70	0.83
	19	0.69	0.98	44.54	0.31	142.50	0.68
	20	0.54	0.99	55.96	0.47	120.08	0.53
**Non-native, n = 696**	AUC = 0.94 (95% CI = 0.91–0.97)						
	11	0.97	0.67	2.93	0.05	58.79	0.64
	12	0.97	0.73	3.56	0.05	77.71	0.69
	13	0.97	0.79	4.67	0.04	110.96	0.76
	**14**	**0.97**	**0.83**	**5.60**	**0.04**	**138.95**	**0.79**
	15	0.87	0.86	6.21	0.15	40.05	0.73
	16	0.80	0.89	7.61	0.22	34.06	0.69
	17	0.73	0.92	8.88	0.29	30.55	0.65

NOTE: AUC = area under the curve; LHR+ = liklihood ratio positive test; LHR− = liklihood ratio negative test; OR = diagnostic odds ratio. All AUC’s were significant (p<0.01). Scores with the highest unroundend Youden’s index are indicated in bold.

**Figure 1:**
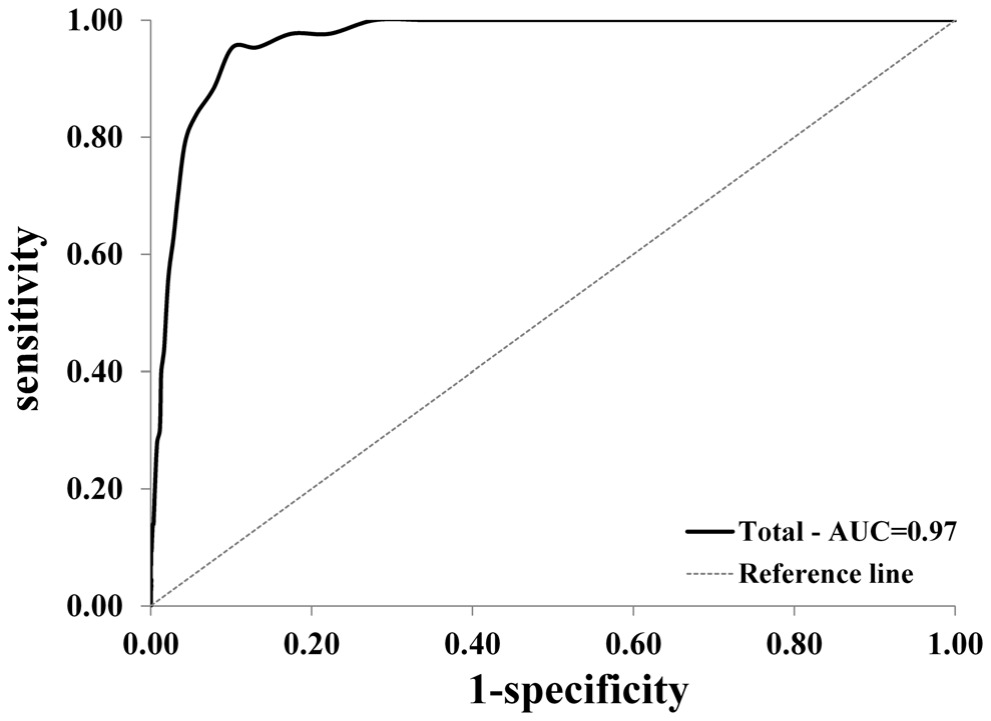
Receiver Operating Characteristic (ROC) curve for BITSEA Problem scores for the total population, relative to CBCL1.5-5 Total Problem score in the clinical range. AUC = area under the curve.

The AUC’s (95% Confidence Intervals: 95% CI) of the Problem scale was for the total population 0.97 (0.95–0.98), for boys 0.95 (0.92–0.98), for girls 0.98 (0.97–0.99), for native children 0.98 (0.97–1.00), for non-native children 0.94 (0.91–0.97). There were no significant differences in AUC between subgroups of gender and ethnicity (i.e. no (unrounded) overlapping confidence intervals). The ROC curve of the BITSEA Problem scale for the total population is presented in Figure 1.

The Youden index indicated the same optimal BITSEA Problem scale cutpoint for boys and girls (score 14), whereas a different optimal cutpoint was indicated by the Youden index for native children (score 17) and non-native children (score 14).

### Clinical Application

Of the 1579 children with complete data of both the parent and the child health professional, child health professionals referred 96 (6.1%) children for further evaluation. Both regression coefficients were significant (p<0.05), BITSEA Problem scale, *B* = 2.5, BITSEA Competence scale, *B* = −0.7. Mean BITSEA scores differed significantly (p<0.05) between children that were referred (Problems scale, M = 10.1, SD = 6.7; Competence scale, M = 17.2, SD = 3.2) and children that were not referred (Problem scale, M = 7.5, SD = 4.9; Competence scale M = 17.9, SD = 2.9). The effect sizes of the differences in mean BITSEA scale scores between children that were and were not referred were for the BITSEA Problem scale *d* = 0.39 and for the BITSEA Competence scale *d* = 0.22. See Table 4.

**Table 4:** Clinical application of the BITSEA; relation between BITSEA scores and the decision to refer to a specialist or request a follow-up consultation, n = 1579.

	Referral or follow-up decision			
BITSEA scales	Mean (SD)		Beta^1^	Effect size^2^
	Not referred	Referred		
	N = 1483	N = 96		
Problem scale	7.5 (4.9)	10.1 (6.6)	2.5	0.39^a^
Competence scale	17.9 (2.9)	17.2 (3.2)	−0.7	0.22^a^

1 Significant unstandardized Beta’s (p<0.05) are corrected for confounding effects of child’s gender and ethnicity.

2 Difference of the means divided by SD in the subgroup ‘referred’ a. indicates a small effect (0.2≤ d <0.5).

Of the children with a score in the clinical range on the Problem scale or Competence scale or CBCL respectively 9.5%; 7.4% and 26.7% were referred.

## Discussion

The present study evaluated the screening accuracy of the BITSEA Problem scale for a large community sample in comparison to a subsample of children with a CBCL Total Problem score in the clinical range. Furthermore, we evaluated the clinical application of the BITSEA Problem and Competence scale. Our results indicate that the BITSEA Problem scale has high screening accuracy and that BITSEA scores were less favourable for children that were referred.

### Screening Accuracy

The BITSEA Problem scale has a good screening accuracy when compared to a group of children with a CBCL Total Problem score in the clinical range (i.e. AUC>90). The study performed in the US [9] found comparable sensitivity for the BITSEA Problem scale as in our study, whereas the specificity in our study was higher.

In our study the Youden index yielded the same optimal cutpoint for boys and girls (score 14). In the US-study, for the age range 24–29 months, score 14 was also identified as the cutpoint for boys, whereas the cutpoint for girls was 13. These different results between studies might be attributed to different characteristics of the study populations and to the different methods of indicating a cutpoint. Also, as opposed to the US-study, in our study completion of the BITSEA was not anonymous, since the answers were used by the child health professional to assess the child’s development.

In our study we found different optimal cutpoints for native and non-native children, where native children differed from the other (sub)samples in cutpoint as indicated by the Youden index; score 17. The mean BITSEA Problem scores differed significantly between boys and girls and native and non-native children, but the difference in mean BITSEA Problem scores between native and non-native children was larger (effect size = (mean_non-native_−mean_native_)/sd_non-native_ = 0.43) than the difference in mean BITSEA Problem scores between boys and girls (effect size  =  (mean_boys_−mean_girls_)/sd_boys_ = 0.15), which might explain the different optimal cutpoints between native and non-native children and not between boys and girls. The outcome that the screening accuracy of the BITSEA is the same for native and non-native children is valuable. However, the application of different cutpoints for different ethnic groups in preventive child health care may not be desirable, since it is difficult to determine whether the different distribution and mean BITSEA scores can be attributed to the actual amount or seriousness of problems, or that it reflects cultural differences (e.g. in interpretation of behavior, or question items). Moreover, the composition of ethnic groups may change over time, which would mean the (continuous) evaluation and adjustment of cutpoints.

The BITSEA Competence scale was excluded from screening accuracy analyses because the content of the items of the BITSEA Competence scale do not resemble the content of the items on the CBCL Total Problem score. This is supported by the low and non-existence of correlations between the mean BITSEA Competence scores and the CBCL Total Problem score found in prior studies [9]–[11]. The decision not to include the BITSEA Competence scale in the analyses seems also (partly) justified by the results of the present study that the median BITSEA Competence score did not differ between girls and native children with and without a CBCL Total Problem score in the clinical range.

### Clinical Application

The BITSEA Problem and Competence score were significant, respectively positively and negatively, associated with the child health professional’s decision whether or not referral was required. These results indicate that scores were less favourable for children who were referred, compared to children who were not referred. However, the difference in mean BITSEA Problem and Competence scores were small (0.20≤d<0.50).

The child health professionals referred 7.4–9.5% of the children with a score in the clinical range on either BITSEA scale and 26.7% of the children that score in the clinical range of the CBCL1.5-5. The frequency of children that were referred was relatively small (n = 96). Moreover, only 30 (2%) children of whom we had complete data of the parent and child health professional, had a score in the clinical range of the CBCL1.5-5 Total Problem score, of which 8 were referred. These small frequencies might have caused a power problem. Other studies found percentages, comparable to our referral frequencies on the CBCL1.5-5. In one study child health professionals referred (or requested a follow-up consultation) 22.4% children with a high score on both the parent and teacher completed SDQ (>P90) [22]. In two other studies child health professionals referred (or requested a follow-up consultation) 19% of the children with a score in the clinical range on the CBCL [23] and ITSEA [24]. However, in these latter two studies the child health professional was blind to the questionnaire score, as were the professionals in our study for the CBCL1.5-5, this might also partly explain the difference in frequencies. Not all children with a score in the clinical range on an early detection instrument are referred, possibly because the problematic emotions or behaviors are mild or are considered to be temporarily (e.g. after a major life event). Then, the child health professional may offer advice about how to cope with the circumstances instead of referring the child to more specialized care [15]. Also, the degree of concerns the parents have about their child’s development is likely to play a role in the clinical decision of the child health professionals, since child health providers are found to be more likely to refer when parents are concerned about their child’s behavior [25], [26].

### Strengths and Limitations

A major strength of our study is that the analyses of screening accuracy were performed on a large and diverse community sample, which adds to the power of the study. Additionally, the answers on the BITSEA were not anonymous, since the child health professional used the BITSEA to assess the child’s development during the well-child visit, this could be seen as either a strength or a limitation. The parents could have completed the BITSEA more seriously; on the other hand it could also have led to more socially desirable answers.

Our study also has some limitations. First, in our sample a low percentage (i.e. 2.1%) of children had a CBCL Total Problem score in the clinical range, whereas based on the literature a higher percentage (i.e. 6.5–12.5%) was expected [23], [24], [27]. This might be indicative of a response bias: not all parents with children with (serious) psychosocial problems may come to the well-child visit, possibly because they are already under treatment of a specialized mental health professional, or because they did not wish to participate in the study. Different cutpoints might be the result of the response bias, as opposed to when the sample consisted of more children with CBCL Total Problem scores in the clinical range. However, the percentage of parents that attended the well-child visit and also completed the questionnaire is quite high (i.e. 95.5%), indicating that the sample is a good reflection of the population in the Rotterdam area that make use of the preventive child health care, may complete the questionnaire in the future and on whom the cutpoints should be applied. However, as a consequence, the subgroups of child gender and ethnicity in the ‘clinical range sample’ are quite small. This does not lead to large confidence intervals since the confidence intervals are calculated based on the large total study population.

Another limitation of our study is the use of children with a CBCL Total Problem score in the clinical range, a subsample of the community sample, as a reference group for the ROC analyses. This excluded the possibility to evaluate the screening accuracy of the BITSEA Competence scale. Moreover, the criterion-related validity of the CBCL (criterion in this case being the presence of psychosocial problems) might limit the quality of findings on screening accuracy. However, the CBCL1.5-5 is a well validated questionnaire and often used as a gold standard for research and clinical work among broad-band early detection instruments for psychosocial problems.

The study was performed in the Netherlands with a Dutch population; this might hamper generalizations to populations of other cultures. However, no difference in screening accuracy was found between native and non-native Dutch children. Moreover, a previous study also showed no difference between native and non-native Dutch children in reliability and validity of the BITSEA [11], suggesting that the BITSEA performs equally well for samples of different cultures.

### Future Research

We recommend future studies to evaluate the screening accuracy of both the BITSEA Problem and Competence scale with a reference group of children with a broad range of psychosocial problems who are diagnosed by a mental health professional. Additionally, evaluating the clinical application of the BITSEA in a larger sample, and including the concerns of parents regarding their child’s development in these analyses, might provide more insight in the value of the BITSEA in the preventive child health care. Furthermore, we recommend evaluating the application of the determined BITSEA cutpoints by child health professionals and the subsequent adherence of the referrals by parents.

### Conclusion

The BITSEA Problem scale shows a good screening accuracy with regard to psychosocial problems as indicated by the CBCL1.5-5, for the total population and for subgroups of child gender and ethnicity. Furthermore, the clinical application of the BITSEA was as hypothesised; less favourable scores for children that were referred, compared to children that were not referred. These results indicate that the BITSEA may be suitable for use in the preventive child health care.
